# Association of Genetic and Allelic Variants of Von Willebrand Factor (VWF), Glutathione S-Transferase and Tumor Necrosis Factor Alpha with Ischemic Stroke Susceptibility and Progression in the Saudi Population

**DOI:** 10.3390/life13051200

**Published:** 2023-05-17

**Authors:** Mohammed M. Jalal, Rashid Mir, Abdullah Hamadi, Malik A. Altayar, Imadeldin Elfaki, Jameel Barnawi, Almohanad A. Alkayyal, Mouminah Amr, Jabali Hadeel, Mamdoh S. Moawadh, Basim S. O. Alsaedi, Marwan H. Alhelali, Aadil Yousif

**Affiliations:** 1 Department of Medical Laboratory Technology, Faculty of Applied Medical Sciences, University of Tabuk, Tabuk 71491, Saudi Arabia; 2 Prince Fahad Bin Sultan Chair for Biomedical Research, Faculty of Applied Medical Sciences, University of Tabuk, Tabuk 71491, Saudi Arabia; 3 Department of Biochemistry, Faculty of Science, University of Tabuk, Tabuk 71491, Saudi Arabia; 4 Neuroscience Center, King Abdullah Medical Complex, Jeddah 23816, Saudi Arabia; 5 Department of Radiology, King Abdullah Medical Complex, Jeddah 23816, Saudi Arabia; 6 Department of Statistics, University of Tabuk, Tabuk 71491, Saudi Arabia; balsaedi@ut.edu.sa (B.S.O.A.);

**Keywords:** ischemic stroke, von Willebrand factor (VWF), glutathione S-transferase (GST), tumor necrosis factor alpha, atherosclerosis, cerebrovascular disease, amplification refractory mutation system (ARMS-PCR)

## Abstract

Stroke is a key cerebrovascular disease and important cause of death and disability worldwide, including in the kingdom of Saudi Arabia (KSA). It has a large economic burden and serious socioeconomic impacts on patients, their families and the community. The incidence of ischemic stroke is probably increased by the interaction of GSTT1 and GSTM1 null genotypes with high blood pressure, diabetes and cigarette smoking. The roles of VWF, GSTs and TNF-alpha gene variations in the induction of stroke are still uncertain and require further examination. In the current study, we studied the associations of SNPs in the genes VWF, GSTs and TNF-alpha with stroke in the Saudi population. Genotyping was performed using the ARMS -PCR for TNF-alpha, AS-PCR for VWF and multiplex PCR for GSTs. The study included 210 study subjects: 100 stroke cases and 110 healthy controls. We obtained significant distributions of VWF rs61748511 T > C, TNF-alpha rs1800629 G > A and GST rs4025935 and rs71748309 genotypes between stroke cases and the healthy controls (*p* < 0.05). The results also indicated that the TNF-alpha A allele was associated with risk of stroke with odd ratio (OR) = 2.22 and risk ratio = RR 2.47, *p* < 0.05. Similarly, the VWF-TC genotype and C allele were strongly linked with stroke with OR = 8.12 and RR 4.7, *p* < 0.05. In addition, GSTT1 and GSTT1 null genotype was strongly associated with stroke predisposition with OR = 8.30 and RR = 2.25, *p* < 0.0001. We conclude that there is a possible strong association between the VWF-T  > C, TNF-alpha G > A, GSTT1 gene variants and ischemic stroke susceptibility in the Saudi population. However, future well-designed and large-scale case–control studies on protein–protein interactions and protein functional studies are required to verify these findings and examine the effects of these SNPs on these proteins.

## 1. Introduction

Stroke is defined as a neurological disorder resulting from an acute focal injury to the central nervous system (CNS) caused by blood vessel blockage, leading to cerebral infarction and hemorrhage in the intracerebral and subarachnoid tissues [1]. Generally, it is classified as ischemic or hemorrhagic: ischemic stroke is because of obstruction of blood vessels, leading to shortage of blood supply to the brain tissues, whereas hemorrhagic strokes are caused by damage to blood vessels, resulting in intracranial hemorrhage [2]. According to the cause, there are five stroke subtypes: atherosclerosis of large artery, cardioembolic, occlusion of small blood vessel, stroke of other known etiology, and stroke of unknown etiology [3]. The etiology of stroke includes hematological disorder, dissection, migraine, cancer, homocysteinemia, infectious arteritis and elevated lipoprotein A [4]. Stroke is a highly prevalent disorder with serious socioeconomic impact, and is an important cause of death and disability [5]. It is ranked as the second-highest cause of death with a mortality rate of 5.5 million deaths yearly, and about half of the survivors present with chronic disability [6]. The risk factors of stoke are classified into nonmodifiable risk factors, including age, sex and ethnicity or race [7], and modifiable risk factors, including high blood pressure, smoking, unhealthy diet, physical inactivity, hyperlipidemia, alcohol consumption and diabetes [7]. Genetic risk factors are classified in between the modifiable and nonmodifiable risk factors, since they are considered as potentially modifiable via modification of the gene–environment interplay [7]. Stroke is classified into two categories. Firstly, ischemic stroke occurs due to closure of the vasculature that supply the brain tissues; secondly, hemorrhagic stroke is caused by blood vessel rupture, leading to intracranial hemorrhage [8]. 

von Willebrand factor (VWF) or factor VIII-related antigen is a large glycoprotein produced by the endothelial cells and megakaryocytes and found in plasma and endothelium [9]. The VWF is very important for hemostasis [9]. It binds factor VIII coagulant protein; this binding results in the activation of factor X in the intrinsic pathway of coagulation [10]. In addition, VWF has important roles in platelet adhesion [10]. Individuals with dysfunctional VWF suffer from a bleeding disorder called von Willebrand disease (VWD) [11]. There are three types of VWD: in Type 1, reduced plasma levels of VWF are seen, and in type 2 VWF is impaired [12]; in type 3, VWF is entirely absent [12]. It has been reported that the VWF has a role in ischemic stroke, and that it can be a target for stroke treatment [13,14]. 

Glutathione S–transferase (GST) is an enzyme with a molecular weight of 26 KDa present in eukaryotic cells [15], is involved in the removal of toxins, and assists in reduction of oxidative stress [16]. It is reported that GST is up-regulated as a mechanism to protect cells from oxidative stress [15,17]. There are eight mammalian GST classes, designated as alpha, mu, pi, omega, sigma, theta, zeta and kappa. The first seven classes are cytosolic [18]. Gene variations in GST have been associated with diseases such as cancers, cardiovascular disease and Alzheimer’s disease [18,19]. 

**Figure 1:**
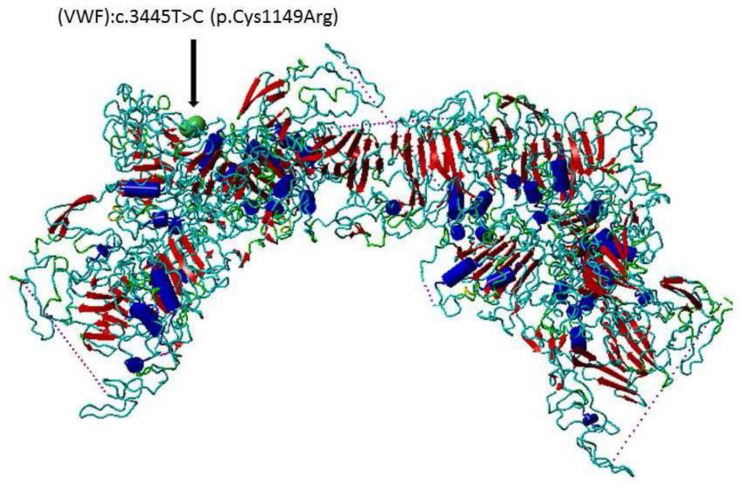
The protein structure of von Willebrand factor (VWF). The secondary dimensional structure (ribbon through) of the von Willebrand factor (VWF), PDB ID 7PMV. The SNP resulted in substitution of Cysteine to Arginine. The site of amino acid substitution is indicated with arrow and surface structure. This figure was prepared with YASARA.

Tumor necrosis factor alpha (TNF-alpha) is a pro-inflammatory and antitumorigenic cytokine that is involved in various autoimmune disorders [20]. TNF-alpha has very important effects on the metabolism and functions of the adipose tissues [20,21], and is secreted by activated macrophages, T-lymphocyte cells and natural killer cells [22]. TNF-alpha has been reported to promote the atherosclerosis in mice [23], and that the activated pathways of TNF-alpha signal transduction have possible roles in induction of atherosclerosis [24], an important cause of stroke. It has been reported that TNF-alpha induces the expression of WBC adhesion molecules, producing oxidative stress, local inflammation, thrombosis and hemorrhage in stroke [21]. In addition, the TNF-alpha levels are increased in the cerebrospinal fluid and serum of stroke cases [21]. In the current study, we examined the associations of the GSTs SNPs (Mu 1 (GSTM1) and Theta 1 (GSTT1)), TNF-alpha SNP rs1800629 G > A SNP and VWF SNP rs61748511 T  > C (Figure 1) with stroke in the Tabuk population. 

## 2. Methodology

### 2.1. Study Population

Only Saudi Arabs were included in the study; all other Arabs, non-Arabs and recently naturalized Arabs were excluded in order to conduct an ethnically preserved genetic variation study.

The study enrolled 220 subjects (105 of which were stroke patients and the remaining 115 of which were healthy counterparts). The specimens were collected from the OPD of the Neurology department at King Khaled hospital and King Salman Military Hospital-Tabuk, while participants visiting the aforementioned hospitals for routine medical checkups provided samples for the healthy control group. The questionnaire and informed consent form were fully completed by the study’s participants. The University of Tabuk’s Ethics Committee granted its clearance for this project (UT-91-23-2020) in accordance with ethical standards.

The study followed the standard protocols of including human subjects in research and met the principles set forth in the Helsinki Declaration. Before obtaining samples from either patients or controls, informed consent was obtained.

### 2.2. Inclusion and Exclusion Criteria for Stroke 

The inclusion criteria comprised stroke cases with clinical confirmation. Stroke patients with only Saudi ethnicity, both male and female stroke patients and of all age groups were included. Stroke patients with a history of any serious clinical conditions were also excluded from the study, as were individuals who were not Arabic.

### 2.3. Inclusion and Exclusion Criteria for Healthy Controls

The inclusion criteria for controls included healthy volunteers who had no history of serious clinical conditions. Both the male and female subjects were of Saudi ethnicity. The control samples did not involve additional phlebotomy because they were timed to coincide with regular blood draws that are a part of a regular check-up. A questionnaire and written informed permission forms were given to each healthy control participant.

### 2.4. Specimen and Data Collection from the Stroke Patients

In an EDTA or Lavender top tube, 3 mL peripheral blood specimen was taken from stroke patients as well as from healthy volunteers. Blood samples were stored immediately at −20 °C. Data were gathered using a standardized questionnaire that contained demographic details such as gender and age. Systemic hypertension was one of the cerebrovascular risk factors, which was indicated by two outpatient blood pressure readings above 140/90 mmHg or by prior antihypertensive medication use for this purpose. Dyslipidemia (Triglycerides > 150 mg/dL LDL > 100 mg/dL, total serum cholesterol > 200 mg, HDL < 50 mg/dL), T2D, Atrial fibrillation.

### 2.5. Genomic DNA Extraction from Stroke Patients and Healthy Controls 

According to the manufacturer’s recommendations, genomic DNA was extracted from samples of patients and healthy groups using a standard DNeasy Blood K (Qiagen-Hilden, Germany). After that, DNA samples were dissolved in nuclease-free water and kept at 4 °C. The purity of the collected DNA was examined by measuring optical density (OD) at A_260_ and A_280_ using NanoDropTM (Thermo Scientific, Waltham, MA, USA). A260/A280 ratios between 1.80 and 1.96 indicated high-quality DNA.

### 2.6. Amplification Refractory Mutation System PCR of TNF-α rs1800629 G > A Genotyping

Amplification Refractory Mutation System PCR (ARMS-PCR) was used to identify the TNF-promoter region polymorphism rs1800629 G/A in stroke and control samples. Primer 3.0 v was used to design the TNF-α rs1800629 G > A primers as depicted in Table 1. The PCR reaction was carried out in a reaction volume of 25 μL comprising template DNA (50 ng), Fo—0.20 μL, Ro—0.20 μL, FI—0.25 μL, RI—0.25 (25 pmol of each primer) and 12 μL from Green PCR Master Mix (2X) Cat M712C (Promega, Madison, WI, USA). Nuclease-free ddH_2_O was added to adjust the final volume to 23 μL. About 2–3 μL DNA was added from each patient. Thermocycling settings included an initial extension at 95 °C for 8 min, followed by 32 cycles of 95 °C for 36 s, 60 °C for 38 s and 72 °C for 34 s, before storage at 4 °C for infinity.

#### Gel Electrophoresis and PCR Product Visualization

Polymerase chain reaction products were processed in 2.5 percent agarose gel which was stained with Sybre safe dye. The Gel Documentation System by Bio-Rad was used to visualize the gel picture of TNF-α rs1800629 G > A genotyping. The TNF-alpha gene’s exon is flanked by the primers Fo and Ro, which yielded a 323 bp band that served as a quality and quantity check for the DNA. A band of 224 bp was yielded by the G allele using primers Fo and RI, whereas a band of 154 bp was yielded by the A allele using primers FI and Ro.

### 2.7. Multiplex PCR for GSTT1 and GSTM1 (rs4025935 and rs71748309) Genotyping 

**Figure 2:**
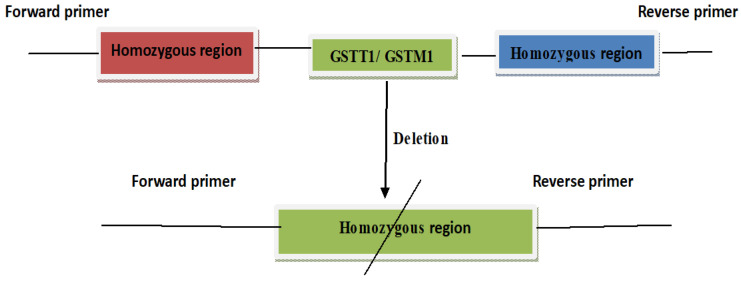
GST gene. GST gene and locations of the primers.

A multiplex PCR assay was optimized for the purpose of simultaneously detecting the allelic status of GSTM1 and GSTT1 (rs4025935 and rs71748309). Six primer pairs were used in the test, which amplified fragments of the GSTM1 and GSTT1 genes, as well as fragments unique to deletions in the GSTM1 and GSTT1 genes. It is thought that an uneven crossover between two extremely homogenous areas at each side of the genes is what causes the GSTM1 and GSTT1 deletions (Figure 2). Long DNA segments must be amplified to produce bands specific for the deletions, since the repetitive sections on either side of the genes have a high degree of similarity. The lengths of the amplifications were kept as short as possible to make the multiplex PCR as robust as possible. To obtain balanced reactions with all conceivable amplicon combinations present, the multiplex PCR was optimized.

The multiplex PCR reaction was performed in a reaction volume of 25 μL comprising template DNA (50 ng), primer of GSTM1-F1—0.25 µL, GSTM1-R1—0.25 µL, GSTT1-F2—0.25 µL, GSTT-R2—0.25 µL (25 pmol of each primer) and 12 µL from Green PCR Master Mix (2×) (Cat# M712C, Promega, Madison, WI, USA), and the final reaction volume of 23 µL was adjusted by adding nuclease-free ddH_2_O.Thermocycling conditions included 10 min at 95 °C followed by 32 cycles of 95 °C for 37 s, 60 °C for 42 s followed by the final extension at 72 °C for 12 min and storage at 72 °C for infinity.

#### Gel Electrophoresis and PCR Product Visualization

Both the GSTM1 and GSTT1 positive genotypes are indicated by the presence of all three band sizes (215 bp, 480 bp and 312 bp). As shown in Figure 1, a single band size of 312 bp shows genotypes that lack both GSTM1 and GSTT1, band sizes of 215 bp and 312 bp, suggesting genotypes that lack both GSTM1 and GSTT1 and band widths of 480 bp and 312 bp, indicating genotypes that lack both GSTM1 and GSTT1, respectively.

### 2.8. Allele-Specific PCR Primers for VWF rs 61748511T > C (VWD c.3445T > C)

Allele-specific PCR was used for VWF rs61748511T > C (VWD c.3445T > C) for genotyping. Table 1 shows the primer sequences of VWF rs61748511T > C.

For C allele: 

AS-PCR reactions were carried out in a reaction volume of 25 µL comprising template DNA (50 ng), VWF-F1—0.25 µL, common reverse VWF-R-0.25 µL (25 pmol of each primer) and 12.5 µL from Green master Mix (Promega, Madison, WI, USA). The final volume of 12 µL was adjusted by adding nuclease-free ddH_2_O.

For T allele: 

The PCR reaction was carried out in a reaction volume of 25 μL comprising template DNA (50 ng), VWF-F2—0.25 µL, common reverse VWF-R-0.25 µL (25 pmol of each primer) and 12.0 µL from Green master Mix. The final volume of 23 µL was adjusted by adding nuclease-free ddH_2_O.

The thermocycling conditions used were at 94 °C for 5 min, followed by 36 cycles of 95 °C for 31 s and 53 °C for VWF rs 61748511T > C for 35 s, which was followed by the final extension at 72 °C for 9 min and storage at 72 °C for infinity.

### 2.9. Statistical Analysis

The Chi-square Hardy–Weinberg equilibrium (HWE) test was used to compare allele frequencies between patients and controls. A *p*-value of 0.05 or less was regarded as significant. SPSS 16.0 was used to perform statistical analysis. The chi-square (2) goodness-of-fit test was used to estimate deviations from the Hardy–Weinberg disequilibrium (HWD). Using the Chi-square test, differences in the genotype frequencies of TNF- rs1800629 G > A, GSTT1 and GSTM1 (rs4025935 and rs71748309), and VWF rs 61748511T > C (VWD c.3445T > C) were assessed. The odds ratios (ORs), risk ratios (RRs) and risk differences (RDs) with 95% confidence intervals (CIs) were computed to estimate the associations between the VWF rs 61748511T > C (VWD c.3445T > C), GSTT1 and GSTM1 (rs4025935 and rs71748309) and TNF-alpha rs1800629 G > A genotypes and risk of stroke patients.

## 3. Results 

### 3.1. Demographic Features of Stroke Patients

Biochemical Characterization: This case–control study was composed of 215 study participants, among which 100 were clinically confirmed cases of stroke patients and 115 were healthy controls. All the demographic information for the 215 stroke patients and healthy control participants who received treatment sequentially is summarized in Table 2. For a total of 100 individuals, full clinical data were available. However, stroke patients were split into two categories based on their ages: those over 45 and those under 45. The biochemical characteristics of the patients and the control group included lipid profiles, hormone profiles and indicators for type-2 diabetes, including free fasting glucose insulin, which are typically altered in stroke patients. Colorimetric estimations (Cobas Integra 800; Roche) were used to create the lipid profile for HDL, TAGs LDL and cholesterol. Using the appropriate ELISA kits, the serum concentrations of several hormones, including progesterone, TSH, FSH, LH, estradiol and testosterone, were measured (Table 2).

### 3.2. Statistical Comparisons between Stroke Patients and Controls (p-Values) for VWF rs61748511 T > C (c.3445T > C, p.Cys1149Arg): TNF-α rs1800629 G > A genotypes

VWF rs61748511, also called c.3445T > C, p.Cysteine1149Argine (C1149R), is a single-nucleotide variation in the VWF gene on chromosome 12. The rare rs61748511(C) allele is regarded as pathogenic for von Willebrand disease. 

Between stroke patients and healthy controls, there was a substantially different distribution of VWF rs 61748511 T/C gene polymorphism genotypes (*p* = 0.017), as depicted in Table 3, while the TT, CT and CC genotype frequencies in healthy individuals were 70%, 28% and 2%, respectively (Table 3). Stroke patients and controls shared a statistically significant (*p* = 0.0001) correlation for VWF rs61748511 T > C genotypes. Moreover, the frequency of the C allele (fC) was found to be significantly higher among patients than in controls (0.48 vs. 0.16). The GG, GA and AA genotype frequencies were 77%, 21% and 2% in stroke patients, respectively, whereas in healthy controls the GG, GA and AA genotype frequencies were 89%, 10.90% and 0%, respectively (Table 3). The association of TNF-α rs1800629 G > A genotypes between stroke patients and controls was statistically significant (*p* = 0.038, Table 3). Moreover, the frequency of the A allele (fA) was found to be significantly higher among stroke cases than in controls (0.13 vs. 0.5).

After adjusting for a number of factors and using healthy controls as a reference group, logistic regression analysis was conducted to establish the adjusted odds ratios (OR) and 95% confidence intervals (CI) related with the susceptibility to stroke.

We discovered an allele-dosage-dependent relationship between an increased risk of stroke and the VWF rs61748511 T > C SNP genotype (Table 4). The TC and TT genotypes of the VWF gene were found to be related with risk and susceptibility to stroke in the codominant model, with OR 8.12 (95%) CI = (4.1752 to 15.8113), RR = 2.58 (1.859 to 3.588) and *p* < 0.0001, while the VWF rs61748511 CC genotype was significantly associated with stroke risk, with OR 26.1 (95%) CI = (5.53 to 124.52), RR = 6.61 (1.79 to 24.41) and *p* < 0.0001, (Table 4). According to the dominant inheritance model, there is a strong correlation between the VWF-TT vs. (TC + CC) genotypes and stroke susceptibility, with an OR = 9.3, (95%) CI (4.8704 to 17.8857), RR = 2.85 (2.0615 to 3.945) and *p* < 0.0001. In the dominant inheritance model, there was reported to be a significant connection between the VWF-TT vs. (TC + CC) genotypes and the risk of stroke, with an OR = 9.3, (95%) CI (4.8704 to 17.8857), RR = 2.85 (2.0615 to 3.945) and *p* < 0.0001. Additionally, we discovered a significant association between the VWF-(TC + TT) and VWF-CC genotypes and risk of stroke in the recessive inheritance model, with OR = 8.38, (95%) CI (1.8626 to 37.7242), RR = 4.48 (1.2115 to 16.612) and *p* < 0.005 (Table 4). In allelic comparison, the VWF C allele was linked to an increased risk of stroke with an OR of 4.7 (95% CI) (2.9718 to 7.5922), RR 2.44 (1.7842 to 3.343) and *p* < 0.0001.

### 3.3. Potential Association of the TNF-Alpha rs1800629 SNP G > A Genotypes with Risk to Stroke

The results indicated a possible connection between the TNF-alpha-GA genotype and enhanced stroke susceptibility in the codominant model, with OR = 2.22, (95%) CI = (1.031 to 4.808), RR = 1.54 (0.9624 to 2.464) and *p* = 0.041 (Table 5). The TNF-GG and (GA + AA) genotypes were strongly associated with an increased risk of stroke in the dominant inheritance model (OR = 2.43, 95% CI (1.1418 to 5.2115), RR = 1.63 (1.0136 to 2.6319) and *p* = 0.021) (Table 5). There was no discernible difference between the TNF-(GG + GA) and TNF-AA genotypes in the recessive inheritance paradigm. Stroke susceptibility: OR = 5.60, 95% CI (0.2660 to 118.266), RR = 3.17 (0.2518 to 39.9659) and *p* = 0.260 (Table 5). Having an OR of 2.47, (95% CI) (1.2088 to 5.072), RR of 1.67 (1.0423 to 2.6901) and *p* = 0.013. In allelic comparison, the TNF-A allele was highly correlated with stroke risk. The TNF-GA and TNF-GG + AA genotypes had a substantial impact on the risk of stroke in the over dominant inheritance model, with OR = 2.17, (95%) CI (1.00065 to 4.682), RR = 1.52 (0.951 to 2.43) and *p* = 0.048 (Table 5).

### 3.4. Potential Association of GSTT1 (+) and GSTT1(−) Genotypes with the Susceptibility to Stroke

To determine the correlation between the GSTT1 (+) and GSTT1(−) genotypes and susceptibility of stroke between the Tabuk population, using 95% confidence intervals, a multivariate analysis based on logistic regression was conducted, such as RR, OR and risk difference, with 95% confidence intervals was calculated for each group. With respect to GSTT1 genotype, it was reported that compared to the presence of the GSTT1 genotype, the OR and RR for the GSTT1 null genotype were estimated to be 8.30 (3.8772 to 17.801), 2.25 (1.7667 to 2.890), with statistically significant differences (*p*  =  0.0001), respectively (Table 6). However, with respect to the presence of GSTM1 genotype, the OR and RR for the GSTM1 null genotype were estimated to be OR = 0.90 (0.4993 to 1.649) and RR = 0.94 (0.6904 to 1.306), with a statistically non-significant difference (*p*  =  0.74), respectively (Table 6). There was no significant association of GSTT1 (+) GSTM1 (+) and GSTT1 (+) GSTM1 (+) both present and both null genotype with stroke risk; OR = 0.57 (0.2867 to 1.155), RR 0.72 (0.4855 to 1.090) *p* < 0.120. No association of GSTT1 (+) GSTM1 (−) and GSTT1 (−) GSTM1 (−) genotypes with stroke risk was reported. 

### 3.5. Association of VWD rs61748511 T/C Genotypes with the Clinical Features of Stroke Patients

Our findings showed a significant correlation between the age of stroke patients and the VWD rs61748511 T > C genotypes (*p* 0.003) (Table 7). However, the results indicated that there is no correlation between the gender of patients with the VWD rs61748511 T > C genotypes (*p* = 0.81). A significant correlation (*p* 0.003) between VWD rs61748511 T > C genotypes and HbA1c% levels in the blood of stroke patients was found by statistical analysis of the data. Our findings demonstrated a (*p* 0.035) substantially significant correlation between the VWD rs61748511 T > C genotypes and HDL-C (mg/dL) of stroke patients. There was no correlation between the VWD rs61748511 T > C genotype and fasting glucose, triglyceride, cholesterol, or LDL-C levels (Table 7).

### 3.6. Association of TNF-α rs1800629 G > A Genotypes with the Clinical Features of Stroke Patients

Clinically significant TNF-alpha rs1800629 G > A genotype distribution was found in both male and female stroke patients (*p* = 0.02). Similar to what our findings showed, there was a significant correlation between the age of stroke patients and the TNF-alpha rs1800629 G > A genotypes (*p* 0.032) (Table 7). TNF-alpha rs1800629 G > A genotypes and fasting blood glucose levels were significantly associated (*p* 0.0001). Our findings, however, demonstrated that there was no correlation between TNF-alpha rs1800629 G > A genotypes and patient HbA1c% (*p* 0.241). There was no correlation between the TNF-alpha rs1800629 G > A genotype and triglyceride, cholesterol, or LDL-C levels. However, there was a substantial difference in the TNF-alpha rs1800629 G > A genotype between individuals with lowered and individuals with normal HDL-C mg/dL (Table 7).

## 4. Discussion

Stroke is an important cerebrovascular disease and is ranked as the second-highest cause of chronic disability and death worldwide, with increasing prevalence [28,29]. The risk factors of stroke include diabetes mellitus, hypertension, smoking, increased blood lipid, overweight, aging and illness such as coronary artery disease, HIV, sickle cell anemia and cerebral malaria [29,30]. Aortic arch atherosclerosis is an important risk factor for ischemic stroke [31]. Lipid deposit hardens the arterial wall and leads to the narrowing or blockage of blood flow via formation of clots. Atherosclerosis of the cerebrovascular system may result in incident stroke [31]. Genome-wide association studies (GWAs) have discovered links between many loci and diseases such as diabetes, CVD and cancers [32,33,34,35]. 

### 4.1. Von Willebrand Factor (VWF) VWF rs61748511 in STROKE

**Figure 3:**
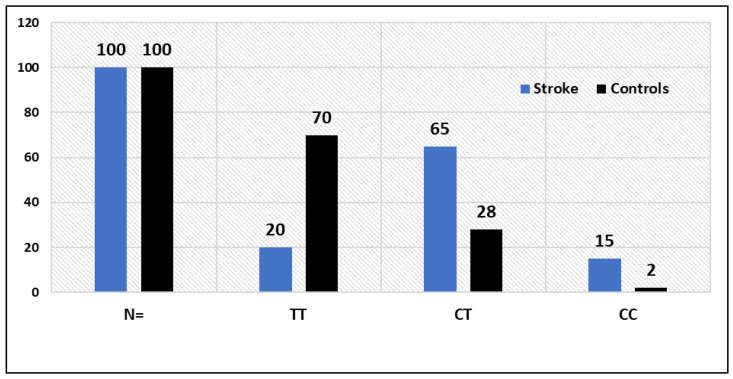
Frequency of VWF rs61748511 T > C genotypes between stroke patients and controls.

von Willebrand disease (VWD) is an inherited hemorrhagic disease caused by von Willebrand factor (VWF) gene mutation. VWF is a glycoprotein function in hemostasis [27], and is secreted by the endothelial cells and megakaryocytes and involved in atherosclerosis [36]. Elevated VWF increases the coagulation and susceptibility to thrombosis [37]. From animal, epidemiological, genetic and patient (with either excess or VWF deficiency) studies, it has been concluded that VWF can be a target for stroke treatment as it plays roles in atherogenesis by bridging vascular collagen and thrombocytes [14]. Furthermore, VWF has been reported to have important roles in thrombocyte adhesion and aggregation at the location of increased damaged rates, such as in coronary arteries with atherosclerosis [38]. Since VWF plays an important role in thrombocyte adhesion and formation of thrombus, it has been reported that there is a positive association of increased levels of VWF and the development of coronary artery disease and stroke [13]. Our results indicated that VWF rs61748511 T  > C SNP was associated with stroke in our cases (Table 3 and Table 4). The results showed that the TC genotype and the C allele of the rs61748511 T  > C SNP were associated with stroke (Table 3 and Table 4). The frequency of VWF rs61748511 T  > C genotypes among stroke and healthy controls is depicted in Figure 3.

Moreover, single-nucleotide variations in VWF gene increase the risk of ischemic stroke and cardiovascular disease [13,39]. The VWF rs61748511 T  > C SNP (VWF c.3445T > C p.Cys1149 Arg) may result in drastic changes, since the sulfur-containing amino acid cysteine (Cys) 1149 is replaced by the positively charged amino acid arginine (Arg) (Figure 1). The influence of this gene variant on the VWF is yet to be investigated in protein functional and protein–protein interaction (PPI) studies [40,41,42,43]. This SNP has been reported to be associated with von Willebrand Disease in a Pakistani population [27]. It has been reported that suppression of VWF in an animal model with atherosclerosis decreases the inflammation and the size of the plaque, as well as the adhesion of platelets [14]. To the best of our knowledge, this is the first study reported the potential association of VWF rs61748511 with stroke development.

### 4.2. Glutathione-S-Transferases (GSTs) in Stroke

There are eight classes of Glutathione-S-transferases in cells, designated as alpha, mu, kappa, omega, pi, sigma, theta and zeta [44]. There are isozymes in every class [44]. GSTs have important roles in the prevention of oxidative stress and many toxic molecules, and play a role in the production of leukotrienes and prostaglandins [45]. The Glutathione S-transferases (GSTs) play roles in the detoxification of free radicals (reactive oxygen species) [46]. The generation of ROS is involved in the development of atherosclerotic disease, such as cardiovascular disease and stroke and cancers [46,47,48]. The free radicals generated from oxidative stress result in lipid peroxidation of the cell membrane and lipoprotein modifications, leading to acceleration of atherosclerosis development [49]. The genetic variations in the Glutathione-S-transferases lead to reduced catalytic enzyme activity that result in increased susceptibility to toxic chemical substances [19]. Gene polymorphism of glutathione S-transferase was reported to be associated with T2D in an Indian population [50]. Our results indicated that the GSTT1 and GSTT1 null genotypes were associated with stroke (Table 3 and Table 6). This result may be consistent with a study reporting the association of GSTT1 alone and GSTM1 and T1 with myocardial infarction and coronary artery disease in Bangladesh and Indian populations [51,52]. This result is in agreement with a study that reported the association of this polymorphism with ischemic stroke in a Chinese Han population [49]. Our results are also consistent with a study reported that the null GSTT1 genotype was associated with increased susceptibility to cerebral stroke in a Russian population [53]. 

### 4.3. Tumor Necrosis Factor Alpha (TNF-Alpha) rs1800629 G > A in Stroke

Our results demonstrated that the TNF-alpha rs1800629 G > A was associated with stroke in our cases (Table 3, Table 4 and Table 5), and that the GA genotype and the A allele of TNF-alpha rs1800629 were associated with stroke development (Table 3, Table 4 and Table 5). The plasma TNF-alpha levels were shown to be significantly increased in stroke cases compared to healthy controls [54], and that suppression of TNF-alpha was suggested to be a therapeutic strategy for stroke [55]. 

The TNF-alpha -308 G  >  A (rs1800629) A allele directly influences the transcriptional activity of the TNF-alpha gene that leads to increased expression of TNF-alpha [56]. This result agrees with a study which demonstrated the association of this SNP with ischemic stroke in a Thai population [57]. TNF-alpha initiates the atherogenesis by increasing the level of reactive oxygen species and reducing the production nitric oxide, which results in endothelial dysfunction [24]. In addition, TNF-alpha contributes to the formation of atherosclerotic plaques via the elevation of low-density lipoprotein transcytosis in the endothelial cells [24]. These lead to atherosclerosis, which is an important cause of stroke [58]. 

**Figure 4:**
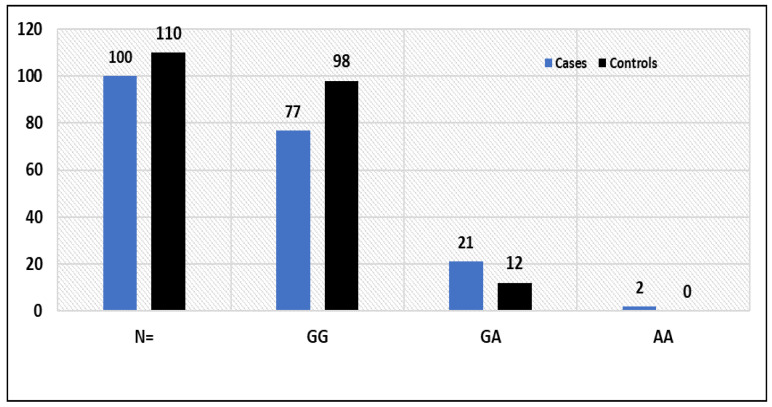
Frequency of TNF-alpha rs1800629 G > A genotypes between stroke patients and controls.

The frequency of TNF-alpha rs1800629 G > A genotypes among stroke patients and healthy controls is depicted Figure 4.

Results also indicated that TNF-alpha rs1800629 G > A genotype distribution was associated with age of stroke patients (Table 7), and that the TNF- alpha rs1800629 G > A genotype distribution was significant different in cases with elevated HbA1c and cases with normal HbA1c (Table 7). This result is in agreement with studies reporting the association of rs1800629 G  >  A with T2D in Chinese and Egyptian populations [59,60,61]. It has been reported that in T2D there are increased concentrations of TNF-alpha in muscle [62], fat tissue and blood, and that elevated levels of TNF-alpha negatively regulate the insulin signaling and whole-glucose homeostasis [62].

The A allele of the TNF- alpha rs1800629 G > A is reported to result in more expression of the TNF-alpha, which results in impaired insulin signaling and T2D [61]. The results also showed that there was a significant difference in the TNF-alpha rs1800629 G > A genotype distribution between stroke patients with normal and stroke patients with reduced HDL-C (Table 7). This result is in agreement with the studies which reported that reduced HDL-C is among the risk factors of stroke and cardiovascular disease [63,64], and that the elevated levels of TNF-alpha are associated with higher risk for atherothrombotic diseases, such as stroke and coronary artery disease [65]. 

To our knowledge, this the first study to demonstrate the potential association of the VWF, GST and TNF-alpha genes and stroke development in the Saudi population. These results (after being verified in further studies) can be used for the detection and stratification of the individuals that are at risk of stroke development by genetic testing for screening diagnosis and prognosis [66]. Since stroke risk factors are classified into modifiable and non-modifiable risk factors [7], the modifiable risk factors, including smoking, hypertension and diabetes, can be controlled, which will result in delay or prevention of this disease [67]. 

## 5. Conclusions

In this study, we evaluated the association of the VWF rs61748511 T  > C, TNF-alpha rs1800629 G > A and GST rs4025935, rs71748309 gene variants with risk of ischemic stroke in the Saudi population. The results demonstrated that there are possible associations between these gene variants and ischemic stroke susceptibility. Future well-designed case–control studies with larger sample sizes on protein—protein interaction and protein functional studies are recommended to examine the effect of these SNPs in these proteins. These results, when confirmed, can be used in genetic testing for the screening, diagnosis and prognosis of stroke.

## Figures and Tables

**Table 1:** The primer sequences of TNF-alpha rs1800629 G > A (−308 G/A), GSTT1 and GSTM1 (rs4025935 and rs71748309) genotyping and VWF rs 61748511T > C (VWD c.3445T > C) genotyping.

Direction	Sequence	AT	PCR Product	Reference
ARMS-primers for TNF-α rs1800629 G > A genotyping				
TNF-alpha Fo	5′-ACC CAA ACA CAG GCCTCAGGACTCAACA-3′	60 °C	323 bp	[25]
TNF-alpha Ro	5′-TGGA GGC AAT AGCTTTTGAGGGGCAGGA-3′			
TNF-alpha FI A	5′-AGTTGGGGACACGCAAGCATGAAGGATA-3′		154 bp	
TNF-alpha RIC	5′-TAGGACCCTGGAGGCTAGACCCCGTACG-3′		224 bp	
Multiplex PCR primers for GSTT1 and GSTM1 (rs4025935 and rs71748309) genotyping				
GSTT1 F	5′-TTC CTT ACT GGT CCT CAC A TCTC-3′	60 °C	480 bp	[26]
GSTT1 R	5′-TCA CGG GAT CAT GGC CAGCA-3′			
GSTM1 F	5′-GAA CTC CCT GAA AAGCTAAAGC-3′		215 bp	
GSTM1 R	5′-GTT GGG CTC AAA TAT ACG GTGG-3′			
CYP1A1F	5′-GAA CTG CCA CTT CAG CT GTCT-3′		312 bp	
CYP1A1R	5′-CAG CTG CAT TTG GAA GTGCTC-3′			
Allele specific PCR primers for VWF rs 61748511T > C (VWD c.3445T > C) genotyping				
AS-PCR for T allele				[27]
F1-T	5′-ACT TGA CAG GCAGGTGCACT-3′	55 °C	213 bp	
C-R	5′-ATT GGT GAC GCCCATAGTCC-3′			
AS-PCR for C allele				
F2-C	5′-ACTT GAC AGG CAGGTGCACC-3′	55 °C	213 bp	
C-R	5′-ATTG GTG ACGCCCATAGTCC-3′			

Abbreviations: TNF-alpha: tumor necrosis factor alpha. GST: Glutathione S transferase. VWF: von Willebrand factor. ARMS-PCR: amplification refractory mutation system PCR. AS-PCR: allele-specific PCR. F: forward primer: R: reverse primer. FI—forward inner primer, RI—reverse inner primer, Fo—forward outer primer, Ro—reverse outer primer.

**Table 2:** Comparative clinical characteristics of the study population.

Characteristic	Controls ^a^	Patients	*p* ^b^
Age	33.50 ± 13.50	55.33 ± 8.12	<0.001
Blood sugar fasting	97.70 ± 4.49	103.70 ± 4.50	<0.001
HbA1c	5.50 ± 0.490	6.802 ± 0.390	<0.001
LDL	116.13 ± 9.70	149.40 ± 32.18	<0.001
Cholesterol	121.40 ± 7.40	169.32 ± 54.13	<0.001
VLDL	26.91 ± 5.80	44.2 ± 12.7	<0.001
HDL	47.31 ± 10.7	26.77 ± 4.22	<0.001
Triglyceride	130.09 ± 8.50	175.8 ± 38.4	<0.001
Platelet count	230.19 ± 78.7	252.15 ± 82.13	0.161

^a^ student’s *t*-test, for continuous variables (variables with a normal distribution); ^b^ values are presented as mean ± standard deviation. *p*-values ≥ 0.05 are considered significant.

**Table 3:** Distribution and association of VWF rs61748511 T > C, TNF-alpha rs1800629 G > A genotypes between patients and controls.

Association of VWF rs61748511 T > C Genotypes between Stroke Patients and Controls									
Subjects	N=	TT	CT	CC	X2	DF	T	C	*p* Value
Stroke	100	20(20%)	65(65%)	15(15%)	52.12	2	0.52	0.48	0.0001
Controls	100	70(70%)	28(28%)	2(2%)			0.84	0.16	
**Association of TNF-alpha rs1800629 G > A genotypes between stroke patients and controls**									
**Subjects**	**N=**	**GG**	**GA**	**AA**	**Df**	**X2**	**G**	**A**	***p* value**
Cases	100	77(77%)	21(21%)	02(2%)	2	6.51	0.87	0.13	0.038
Controls	110	98(89%)	12(10.90%)	0(0%)			0.95	0.5	

Abbreviations: VWF: von Willebrand factor. TNF-alpha: tumor necrosis factor alpha. Logistic regression analysis of VWF rs61748511 T > C genotypes to calculate the risk of stroke risk.

**Table 4:** Association of VWF rs61748511 T > C gene polymorphism with the risk of stroke.

Genotypes	Healthy Controls N = 100	Stroke Cases N = 100	Odd Ratio OR (95% CI)	Risk Ratio RR (95% CI)	*p*-Value
Codominant inheritance model					
VWF-TT	70	20	1 (ref.)	1 (ref.)	
VWF-TC	28	65	8.12(4.1752 to 15.8113)	2.58(1.859 to 3.588)	0.0001
VWF-CC	02	15	26.1(5.5333 to 124.529)	6.61(1.7900 to 24.417)	0.0001
Dominant inheritance model					
VWF-TT	70	20	1 (ref.)	1 (ref.)	
VWF-(TC + CC)	30	80	9.3(4.8704 to 17.8857)	2.85(2.0615 to 3.945)	0.0001
Recessive inheritance model					
VWF-(TC + TT)	98	85	1 (ref.)	1 (ref.)	
VWF-CC	02	15	8.38(1.8626 to 37.7242)	4.48(1.2115 to 16.612)	0.005
Allele			1 (ref.)	1 (ref.)	
VWF-T	168	105			
VWF-C	32	95	4.7(2.9718 to 7.5922)	2.44(1.7842 to 3.343)	0.0001
Over-dominant inheritance model					
VWF-(TT + CC)	98	85	1 (ref.)	1 (ref.)	
VWF-(TC)	28	65	2.67(1.5757 to 4.5463)	1.75(1.2488 to 2.460)	0.0003

VWF: von Willebrand factor.

**Table 5:** Association of TNF-alpha rs1800629 G > A gene polymorphism with the risk of stroke susceptibility.

Genotypes	Healthy Controls (N = 100)	Stroke Cases (N = 100)	Odd Ratio OR (95% CI)	Risk Ratio RR (95% CI)	*p* Value
Codominant inheritance model					
TNF-alpha-GG	98	77	1 (ref.)	1 (ref.)	
TNF-alpha-GA	12	21	2.22(1.031 to 4.808)	1.54(0.9624 to 2.464)	0.041
TNF-alpha-AA	00	02	6.35(0.300 to 134.314)	3.35(0.266 to 42.312)	0.23
Dominant inheritance model					
TNF-alpha-GG	98	77	1 (ref.)	1 (ref.)	
TNF-alpha (GA + AA)	12	23	2.43(1.1418 to 5.2115)	1.63(1.0136 to 2.6319)	0.021
Recessive inheritance model					
TNF-alpha -(GA + GG)	110	98	1 (ref.)	1(ref.)	
TNF-alpha-AA	00	02	5.60(0.2660 to 118.266)	3.17(0.2518 to 39.9659)	0.260
Allele			1 (ref.)	1 (ref.)	
TNF-alpha-G	208	175			
TNF-alpha-A	12	25	2.47(1.2088 to 5.0724)	1.67(1.0423 to 2.6901)	0.013
Over-dominant inheritance model					
TNF-alpha-(GG + AA)	98	79	1 (ref.)	1 (ref.)	
TNF-alpha (GA)	12	21	2.17(1.0065 to 4.682)	1.52(0.9513 to 2.437)	0.048

Abbreviations: TNF-alpha: tumor necrosis factor alpha.

**Table 6:** Genotype frequencies [*n* (%)] of GSTT1 and GSTM1 in case and control groups.

Variables			Controls (110)	Cases (100)	
Genotype frequencies of GSTT1 in case and control groups					
GSTT1 (+)			48(43.63%)	10 (10%)	
GSTT1 (−)			52(47.27%)	90 (90%)	
Genotype frequencies of GSTM1 in case and control groups					
GSTM1(+)			28(25.45%)	33 (33%)	
GSTM1(−)			72(65.45%)	77 (77%)	
Genotype frequencies of GSTT1/GSTM1 in case and control groups					
GSTT1 (+) GSTM1 (+)			24(21.81%)	43(38.18%)	
GSTT1 (−) GSTM1 (−)			32(29.09%)	33(30%)	
GSTT1 (+) GSTM1 (−)			24(21.81%)	13(11.81%)	
GSTT1 (−) GSTM1 (+)			20(18.81%)	11(10%)	
Association of GSTM1/GSTT1 null genotypes with stroke risk					
Association of GSTT1 (+) and GSTT1 (−) genotypes with stroke risk					
Variables	110	100	OR (95% CI)	RR (95% CI)	*p* value
GSTT1 (+)	48(48%)	10 (10%)	Ref. 1.00	Ref. 1.00	
GSTT1 (−)	52(52%)	90 (90%)	8.30(3.8772 to 17.801)	2.25(1.7667 to 2.890)	0.0001
Association of GSTM1 (+) and GSTM1 (−) genotypes with stroke risk					
Variables	110	100	OR (95% CI)	RR (95% CI)	
GSTM1 (+)	28(28%)	33 (33%)	Ref. 1.00	Ref. 1.00	
GSTM1 (−)	72(72%)	77 (77%)	0.90(0.4993 to 1.649)	0.94(0.6904 to 1.306)	0.74
Association of GSTT1 (+) GSTM1 (+) and GSTT1 (−) GSTM1 (−) genotypes with stroke risk					
Variables	110	100	OR (95% CI)	RR (95% CI)	*p* value
GSTT1 (+) GSTM1 (+)	24	43	Ref. 1.00	Ref. 1.00	
GSTT1 (−) GSTM1 (−)	32	33	0.57(0.2867 to 1.155)	0.72(0.4855 to 1.090)	0.120
Association of GSTT1 (+) GSTM1 (−) and GSTT1 (−) GSTM1 (−) genotypes with stroke risk					
Variables	110	100	OR (95% CI)	RR (95% CI)	*p* value
GSTT1 (+) GSTM1 (−)	24	13	Ref. 1.00	Ref. 1.00	
GSTT1 (−) GSTM1 (+)	20	11	1.01(0.3741 to 2.755)	1.0(0.7066 to 1.430)	0.97

Abbreviations: GST: Glutathione S transferases.

**Table 7:** Association of TNF-α rs1800629 G > A genotypes with the risk of clinical features of stroke patients.

Clinical Feature		N = 100	GG77	GA21	AA02	X2	DF	*p*-Value
Gender	Male	62	49	12	01	0.42	2	0.81
	Female	38	28	09	01			
Age	Age < 50	20	17	01	02	11.26	2	0.003
	Age > 50	80	60	20	00			
Fasting glucose mg/dL	<100	70	57	12	01	2.63	2	0.26
	>100	30	20	09	01			
HbA1c %	>6	22	11	10	01	11.6	2	0.003
	<6	78	66	11	01			
Triglycerides mg/dL	<200	70	56	13	01	1.31	2	0.51
	>200	30	28	08	01			
Cholesterol mg/dL	<200	82	66	21	01	3.7	2	0.157
	>200	18	11	06	01			
LDL-C mg/dL	<100	75	59	15	01	0.92	2	0.631
	>100	25	18	06	01			
HDL-L mg/dL	<55	19	11	08	0	6.56	2	0.037
	>55	81	66	13	02			
Association of VWD rs61748511 T/C genotypes with respected to clinical features of stroke								
Clinical feature		N	TT 20	TC 65	CC 15	X2	DF	*p*-value
Gender	Male	62	17	02	01	7.7	2	0.020
	Female	38	60	20	00			
Age	Age < 50	20	07	08	05	6.8	2	0.032
	Age > 50	80	13	57	10			
Fasting glucose mg/dL	<100	70	09	55	06	18.9	2	0.0001
	>100	30	11	10	09			
HbA1c %	>6	22	7	13	2	2.78	2	0.241
	<6	78	13	52	13			
Triglycerides mg/dL	<200	69	14	45	10	0.05	2	0.97
	>200	31	06	20	05			
Cholesterol mg/dL	<200	82	06	08	04	4.14	2	0.126
	>200	18	14	57	11			
LDL-C mg/dL	<100	75	14	51	10	1.24	2	0.532
	>100	25	06	14	05			
HDL-C mg/dL	<55	19	06	09	04	3.27	2	0.19
	>55	81	14	56	11			

Abbreviations-LDL-C: low-density lipoprotein cholesterol. HDL-C: high-density lipoprotein cholesterol. HbA1c: hemoglobin A1c.

## Data Availability

All data pertaining to patients and results are available in Molecular Biology Lab, PFBSRC, FAMS, University of Tabuk.
